# Celiac Crisis: A Life-Threatening Complication of Celiac Disease

**DOI:** 10.34172/mejdd.2024.393

**Published:** 2024-10-30

**Authors:** Pooja Soni, Priya Gogia, Rajkumar Kundavaram

**Affiliations:** ^1^Department of Pediatrics, Employees’ State Insurance Corporation (ESIC) Medical College and Hospital, Faridabad, Haryana, India; ^2^Department of Pediatrics, All India Institute of Medical Sciences (AIIMS), Bhopal, Madhya Pradesh, India

**Keywords:** Celiac disease, Celiac crisis, Gluten-free diet, Anti-tissue-transglutaminase antibody (anti-tTG antibody), Hypoproteinemia

## Abstract

Celiac disease (CD) is an immune-mediated enteropathy with varied systemic involvement and association with increased morbidity and mortality. Strong clinical suspicion is the key, and diagnosis is made using histopathology and serology. Though the consumption of a strict gluten-free diet can improve symptoms and limit mucosal damage, curative therapy is still lacking. Significant clinical improvement can be seen after treatment with immunosuppressive therapy; however, there is no definitive role of immunosuppression in preventing complications. Celiac crisis (CC), a serious and life-threatening complication of CD, is characterized by acute onset and rapid progression of gastrointestinal manifestations associated with metabolic and electrolyte disturbances and neurological and renal dysfunction. Management comprises urgent hospitalization, fluid resuscitation, correction of electrolyte imbalance, and albumin infusion. Early identification and diagnosis of CD and timely initiation of a gluten-free diet with proper compliance are of paramount importance in preventing complications, including CC. Regular follow-up after diagnosis is a good approach to assessing adherence to the gluten-free diet, disease activity, and screening for complications. With the advent of improved diagnostic facilities and access to the health care system, timely diagnosis, and efficient management, prognosis has improved significantly in recent years.

## Introduction

 Celiac disease (CD) is a chronic systemic immune-mediated disease occurring in genetically predisposed individuals after exposure to gluten-based diets (containing wheat, rye, and barley) and is characterized by various gastrointestinal (e.g., diarrhea, constipation, vomiting, abdominal distension, abdominal pain, anorexia, weight loss, failure to thrive) and extra-intestinal manifestations (e.g., anemia, delayed puberty, amenorrhea, irritability, fatigue, dermatitis herpetiformis, neurological disorders, arthritis/arthralgia, hypertransaminasemia, osteopenia/osteoporosis, aphthous stomatitis, short stature, endocrinopathies) along with presence of disease-specific autoantibodies and small intestinal enteropathy.^1-5^ Prevalence of biopsy-confirmed CD is estimated to be 0.7%, and seropositivity is 1.4% globally, with a gradual increase in the last few years because of improved knowledge and increased autoimmunity.^1,6^ Estimated incidence of CD is 1% in children.^7^ As discussed by Lebwohl and Rubio-Tapia in their review, in India, the prevalence of CD varies from region to region, and it is greater in the northern region than in southern India (0.1%).^8^

 Apart from gastrointestinal and extra-intestinal presentations of CD, one life-threatening complication of CD, especially in children, is celiac crisis (CC), which is characterized by an acute onset with rapidly progressing gastrointestinal manifestations associated with metabolic and electrolyte disturbances, neurological dysfunction and renal impairment. It can manifest as initial presentation of a previously unrecognized CD or as a consequence of non-compliance to gluten-free diet in a known case of CD.^1^ With the increasing prevalence of CD, the incidence of CC is also gradually increasing, necessitating an increase in awareness for the same due to the life-threatening but salvageable complications of the disease.

## Review

###  Epidemiology 

 The first description of CC in children was by Anderson and Di Sant’agnese in 1953 in the USA, who reported 35 patients with CC in their retrospective analysis.^9^ The exact incidence of CC is not known; however, Babar et al^10^ reported its incidence as 5% in 2011, while Baranwal et al showed rare occurrence due to early diagnosis, infection control, and effective therapy of CD.^11^ Jamma et al estimated CC to be 1% of all CD cases in adults.^12^

 Waheed et al^13^ conducted a cross-sectional study including 126 pediatric patients with CC in developing country Pakistan and reported the male: female ratio to be 1:1.3, with the mean age of presentation being 5.25 ± 1.18 years (1.8-8 years). Consanguinity was noted in 96.77%, and other risk factors for CC were > 3 diarrheal episodes/year (89.52%), early weaning with a gluten-containing diet (93.54%), and poor social status.

###  Pathophysiology 

 The pathophysiology of the occurrence of CC is still unclear, though a combination of an immune stimulus like a gluten-containing diet, infection, and mucosal inflammation can initiate the crisis in the setting of disturbed intestinal motility due to CD.^12^

**Figure 1:**
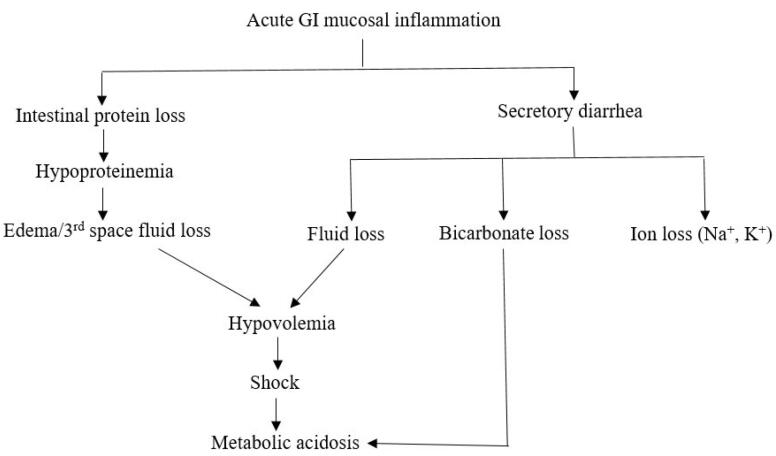
Pathophysiology of celiac crisis

 There are certain triggering factors for the occurrence of CC like infections, previous surgical intervention, pregnancy, hypoproteinemia, lack of compliance to a gluten-free diet in the setting of CD, anticholinergic drugs (atropine sulfate, diphenoxylate hydrochloride). These triggering factors are found in 50% of the patients presenting with CC.^12,14,15^ These factors possibly trigger the activation of the immune system with severe inflammation of the bowel mucosa and a perturbation of normal bowel motility.^12^ A systematic review done by Balaban et al involving 42 adult cases related to CC described precipitating factors in 11 cases.^16^ These included one patient each with trauma, pancreatitis, childbirth, and Bell’s palsy. Three patients had previous surgery, and four patients had infections (Clostridium difficile colitis, herpes simplex esophagitis, urinary tract infection, cytomegalovirus infection). The pathophysiology of CC is depicted in Figure 1.

###  Clinical Manifestations 

 CC generally presents in early childhood, but it may also present in adult and elderly age groups. The mean age of CC onset may also vary with geographical locations. The mean age of onset in developing countries is five years, while in developed and high-income countries, a higher incidence of CC occurs in the first two years of life.^1,17^ CC may occur as a complication of unidentified CD or as a result of non-adherence to the gluten-free diet. As shown by Waheed et al in their study of 126 pediatric patients with CC, five patients were already diagnosed with CD but were poorly compliant with gluten-free diet, while the remaining 121 patients were previously undiagnosed and first time presented with CC.^13^ The most common presentation of CC is profuse diarrhea, leading to dehydration or hypotension, muscle weakness, and edema.^15,18^ Other symptoms include poor feeding, vomiting, abdominal pain, abdominal distension, lethargy, and failure to thrive.^14^ Rare manifestations of CC include ataxia, myoclonus, seizure, encephalopathy, acute kidney injury, hematological changes like thrombocytopenia, and pericardial effusion.^19-21^ In a case reported by Mehta, a 14-year-old girl diagnosed with CC had associated gastrointestinal bleeding due to thrombocytopenia and coagulopathy and, later on, died of pulmonary hemorrhage.^19^ Coagulopathy in CC is attributed to vitamin K deficiency due to malabsorption, which results in its deficiency and prolongation of prothrombin time.^18,22^ Chakrabarti et al reported a 13-year-old child diagnosed with CC, presenting with diarrhea and hypokalemic quadriparesis, which completely subsided after receiving fluids, potassium supplementation, gluten-free diet, and other supplements.^23^ Without management, the condition may worsen with cardiac arrhythmias, neuromuscular weakness, tetany, seizures, acute kidney injury, circulatory collapse, and death.^21,24,25^

 A systematic review done by Balaban et al involving 42 cases presented in 29 articles related to CC in adults, revealed all involved patients had diarrhea at presentation, and significant weight loss was documented in two-thirds.^16^ The clinical picture included altered hemostasis (thrombosis or bleeding) in four patients, neurological manifestations (tetany, paresthesia, neuropathy, limb weakness) in six patients, and cardiovascular involvement (firing of an implantable cardiac defibrillator) in one patient.

 A cross-sectional study of 126 pediatric patients with CC conducted by Waheed et al^13^ revealed 100% of patients had diarrhea at presentation, dehydration in 96.77%, loss of head control in 96.7%, polyuria in 95.96%, abdominal distension in 85.86%, rickets in 72.55%, inability to walk in 67.74%, clubbing in 65.56%, edema in 57.27%, hypotension in 49.2%, 24% had vomiting, and tetany in 4.03%. All patients had weight and height below the 5^th^ percentile for age. Metabolic acidosis was found in 96% of patients.

###  Diagnostic Criteria for Celiac Crisis 

 As proposed by Jamma et al^12^ for adults, diagnostic criteria for CC comprises of acute onset or rapid progression of gastrointestinal symptoms attributable to CD requiring hospitalization and/or parenteral nutrition along with at least two of the following:

- Severe dehydration, including hemodynamic instability and/or orthostatic changes
- Metabolic acidosis (pH < 7.35)
- Dyselectrolytemia, including hyponatremia/hypernatremia, hypokalemia, hypocalcemia, or hypomagnesemia
- Hypoproteinemia (albumin level < 3 g/dL)
- Weight loss ( > 4.5 kg or 10 lb)
- Renal dysfunction (creatinine level > 2.0 g/dL)
- Neurological dysfunction

Though these diagnostic criteria need to be fulfilled for the diagnosis of CC in adults, no criteria have yet been clarified for the pediatric population for diagnosis of CC.^18^

## Investigations

 A known case of CD presenting with acute, severe, and rapidly progressive gastrointestinal symptoms or a previously undiagnosed patient presenting with these symptoms (after ruling out infectious etiologies) with acute metabolic deterioration should be considered for confirmation of CD and workup for CC.^10,13^

 Biochemical abnormalities in CC include hypokalemia, which is the key feature, hyponatremia or hypernatremia, hypophosphatemia, hypocalcemia, hypomagnesemia, hypoproteinemia, hypoalbuminemia, and metabolic acidosis. Other laboratory findings in a case of CC may include anemia, low ferritin, leukopenia, thrombocytopenia, deranged coagulation profile, deranged renal function test, and elevated levels of transaminases.^7,19,26^

 Screening for vitamin (e.g., vitamin B12, folate, vitamin D) and minerals (e.g., iron, calcium, zinc, copper) deficiency should be done in patients presenting with CC since consequences of prolonged deficiencies can lead to significant morbidity.^26^

 For confirmation of CD, auto-antibodies against tissue transglutaminase (anti-tTG - IgA), total immunoglobulin A (IgA), anti-endomysium antibody (IgA), deamidated gliadin peptide (DGP) IgA or IgG, antigliadin antibodies IgA or IgG are used.^5^ Along with the serological confirmation, gastrointestinal endoscopy shows scalloping of duodenal mucosa and intestinal histopathological changes like flattening of the mucosa, villous atrophy, crypt hyperplasia, infiltrative inflammatory lesions with intraepithelial lymphocytes (marsh stage 3 or 4) confirm the diagnosis of CD.^27^

 In a systematic review done by Balaban et al, anti-tissue transglutaminase antibody (anti-tTG) was done in 23 cases with 87% positivity, and antiendomysial antibodies were positive in 15 out of 17 patients with CC (88.2%). Intestinal biopsy was done in all adult CC cases, and all were of Marsh stage 3 or 4.^16^

 Tokgoz et al reported an association of CC and HLA DQB1*0201 in a 13-month-old child (heterozygous) in association with previous literature showing concomitance of homozygous HLA DQB1*0201 with late-onset, more severe clinical manifestations, severe atrophy of villi, severe diarrhea, and lower levels of hemoglobin.^28,29^ Similarly, Radlovic et al reported six children with CC who were homozygous carriers of HLA-DQ2.5 haplotype, suggesting HLA mapping in patients presenting with CC for association of same and future advancement in knowledge.^17^

###  Differential Diagnosis 

 Various differential diagnoses for CC are as follows^1,30^:

1. Infections: viral gastroenteritis, post-infectious gastropathy, tropical sprue, bacterial overgrowth syndrome, AIDS enteropathy, Whipple disease, parasitic infestation, helicobacter pylori-positive gastritis and peptic duodenitis
2. Non-responding CD
3. Drugs: non-steroidal anti-inflammatory drugs, antineoplastic agents, immune modulatory drugs, angiotensin receptor blockers
4. Immune-inflammatory conditions: autoimmune enteropathy, Crohn’s disease, ulcerative colitis-associated duodenitis, eosinophilic gastroenteritis, food protein sensitive enteropathies (allergies to chicken, cow’s milk, eggs, fish, soy), collagenous sprue, immunodeficiencies (including common variable immunodeficiency)
5. Others: Pancreatic insufficiency, laxative use, intestinal lymphoma

###  Management

 Management of CC comprises urgent hospitalization and management of profuse diarrhea, fluid resuscitation, correction of electrolyte imbalance, and albumin infusion to prevent clinical deterioration.^21,24,25,30^ Fluid resuscitation and correction of electrolyte disturbance are life-saving modalities for the CC.^18^ Parenteral nutrition may be required due to massive malabsorptive status, and enteral nutrition with a gluten-free diet should be started as soon as possible.^4^ It is necessary to gradually increase the daily calories to avoid the risk of re-feeding syndrome after initiation of enteral nutrition, which can be fatal if not recognized and appropriately treated.^7^

 The role of corticosteroids in CC is still controversial. A drastic response to steroids in CC has been shown in literature by reducing intestinal inflammation and cell death, promptly reversing mucosal damage, and restoring brush border enzymes.^1,31-34^ Various authors have reported the beneficial role of steroids. Mones et al described two pediatric cases of CC being successfully treated with steroids.^15^ Jamma et al reported rapid clinical improvement within 2 weeks of corticosteroid therapy in adult patients and suggested the use of a brief course of prednisone or budesonide when standard therapy does not result in rapid improvement.^12^ Alharbi showed a clinical response with methylprednisolone 2 mg/kg/d for 5 days in a 3-year-old child with CC after failure to improve with the gluten-free diet.^35^ Hijaz et al achieved complete neurological recovery after giving methylprednisolone 2 mg/kg per day for a 2-year-old child with CC presenting with status epilepticus followed by encephalopathy.^21^ But, few case reports have also shown no role of steroids, as in a 50-year-old patient reported by Shammeri and Duerksen, presenting with CC despite being on corticosteroid (prednisone 30 mg/d) and azathioprine (100 mg/d) for autoimmune hepatitis for 2 months, raising question on the role of modest immunosuppression in prevention of such complication.^36^ Gupta and Kapoor reported two cases of deterioration on steroid therapy.^37^ Moreover, steroid therapy may increase electrolyte depletion by causing kaliuresis, facilitating the occurrence of re-feeding syndrome.^31,37^

 Still, based on the above data available, corticosteroids may be used in cases of CC not responding to supportive therapy and dietary modification or in critical patients, though dose, duration, and type of steroid to be used remains controversial, as seen in previous literature.^11,15,28,38^

###  Prognosis 

 Anderson and Di Sant’agnese reported a 9% mortality rate due to CC in the pediatric age group in 1953.^9^ Since then, very few mortalities have been reported in both adults and children, most likely due to improved diagnostic facilities and health care systems, leading to timely and efficient management of such fatal conditions and increasing awareness, availability, and use of gluten-free diets.

###  Prevention 

 Early identification and diagnosis of CD and timely initiation of a gluten-free diet with proper compliance are paramount important to avoid such a fatal crisis. Screening for CD is recommended for first-degree relatives of patients with CD by anti-tTG IgA and total IgA.^7^ Despite significant clinical improvement after treatment with immunosuppressive therapy, there is no definitive role of immunosuppression in its prevention.^36^

## Conclusion

 CC, a fatal but preventable complication of CD, requires awareness and clinical suspicion for early diagnosis and timely management of life-threatening consequences. Proper compliance with the gluten-free diet is a must for the prevention of such complications. Preventive therapy using immunosuppression is not yet recommended.
